# Cell-Free DNA Fragmentomics in Liquid Biopsy

**DOI:** 10.3390/diagnostics12040978

**Published:** 2022-04-13

**Authors:** Spencer C. Ding, Y.M. Dennis Lo

**Affiliations:** 1 Centre for Novostics, Hong Kong Science Park, Pak Shek Kok, New Territories, Hong Kong, China; dingchen.spencercpy@link.cuhk.edu.hk; 2 Li Ka Shing Institute of Health Sciences, The Chinese University of Hong Kong, Shatin, New Territories, Hong Kong, China; 3 Department of Chemical Pathology, The Chinese University of Hong Kong, Prince of Wales Hospital, Shatin, New Territories, Hong Kong, China; 4 State Key Laboratory of Translational Oncology, The Chinese University of Hong Kong, Prince of Wales Hospital, Shatin, New Territories, Hong Kong, China

**Keywords:** cell-free DNA, NIPT, liquid biopsy, cancer detection, fragmentomics

## Abstract

Cell-free DNA (cfDNA) in bodily fluids has rapidly transformed the development of noninvasive prenatal testing, cancer liquid biopsy, and transplantation monitoring. Plasma cfDNA consists of a mixture of molecules originating from various bodily tissues. The study of the fragmentation patterns of cfDNA, also referred to as ‘fragmentomics’, is now an actively pursued area of biomarker research. Clues that cfDNA fragmentation patterns might carry information concerning the tissue of origin of cfDNA molecules have come from works demonstrating that circulating fetal, tumor-derived, and transplanted liver-derived cfDNA molecules have a shorter size distribution than the background mainly of hematopoietic origin. More recently, an improved understanding of cfDNA fragmentation has provided many emerging fragmentomic markers, including fragment sizes, preferred ends, end motifs, single-stranded jagged ends, and nucleosomal footprints. The intrinsic biological link between activities of various DNA nucleases and characteristic fragmentations has been demonstrated. In this review, we focus on the biological properties of cell-free DNA unveiled recently and their potential clinical applications.

## 1. Introduction

**Figure 1:**
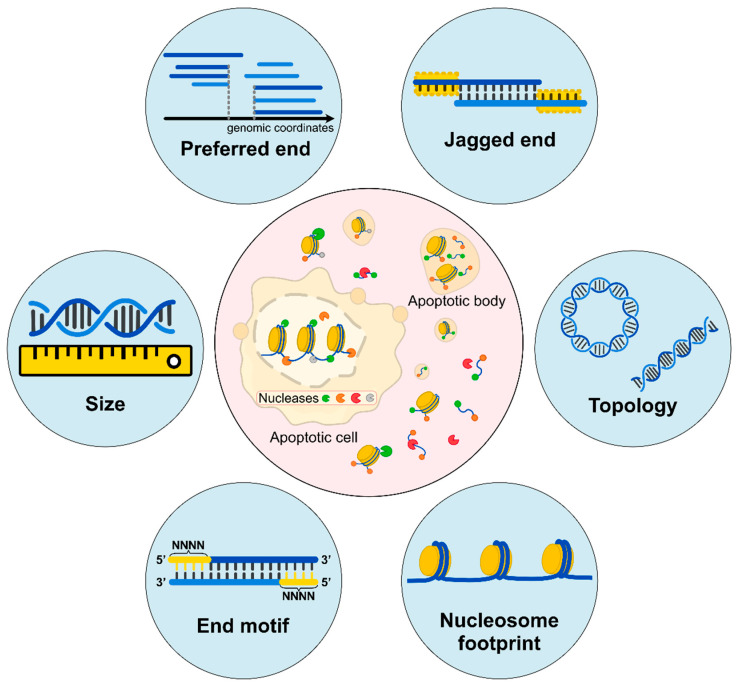
**Biological characteristics of cell-free DNA. (Inner circle)** Apoptotic cells would release DNA molecules into blood circulation accompanied by the digestion of intracellular Deoxyribonuclease 1 Like 3 (DNASE1L3), DNA Fragmentation Factor Subunit Beta (DFFB), and other nucleases. The released cfDNA molecules are further fragmented by extracellular DNASE1L3, Deoxyribonuclease 1 (DNASE1), and other nucleases. Different ends may represent characteristics of cleavages by various nucleases. **(Outer circle)** Fragment sizes are deduced by sequencing in single-base resolution; preferred ends are a subset of the genomic coordinates which are preferentially cleaved; jagged ends are single-stranded ends carried by these double-stranded DNA molecules; DNA topology consists of different forms of DNA molecules, including circular and linear forms; nucleosome footprints show different positioning varies among cell types; end motifs refer to several bases of characteristic sequences at the 5’ end of a fragment (e.g., the 4-nucleotide motif). All these characteristics are reflective of diverse fragmentation processes.

Liquid biopsy has created paradigm shifts in diagnostics and management, including noninvasive prenatal testing (NIPT) [1,2] and oncology [3,4,5,6]. It is notable that a rapid global success in NIPT using cfDNA in maternal plasma has promoted the parallel achievement for cancer detection. To push the frontiers of this field, many efforts have been made to investigate the biological properties of cfDNA molecules in the prenatal and oncology contexts. An improved understanding of the biological characteristics of cfDNA often catalyzes new diagnostic tools. For instance, the realization of the size difference between maternal and fetal DNA leads to a novel approach for detecting fetal chromosomal aneuploidies using fragment sizes [7]. The synergistic use of the size-based and count-based approaches could improve the accuracy of data interpretation, such as the fetal/maternal origin of copy number alterations seen in maternal plasma [8]. Similarly, the characteristic size profiles of tumor-derived DNA molecules could aid in cancer detection [5,9,10]. With the advent of new sequencing technologies and bioinformatics tools, significant progress has been made in understanding the biological characteristics of cfDNA molecules. This review focuses on the properties of cell-free DNA unveiled to date, including fragment sizes, end motifs, preferred ends, nucleosome footprints, jagged ends, and DNA topology, together with discussions on diagnostic implications (Figure 1).

## 2. Sizes of cfDNA Molecules

### 2.1. Methods for Characterizing cfDNA Sizes

The size of cfDNA molecules was previously revealed in gel electrophoresis. Ladder-like bands displaying the multiples of ~180 bp [11,12,13] resembled apoptotic DNA fragmentation. Electrophoresis has relatively low resolution and is not able to precisely measure the sizes of cfDNA molecules. Researchers attempted to use a quantitative polymerase chain reaction (qPCR) assay to reflect the relative amount of cfDNA molecules across different size ranges by designing amplicons with different sizes [14,15,16]. For example, the relative readouts between different sized amplicons would indicate the relative quantities of cfDNA molecules across different sizes. Chan et al. used this principle to reveal that the fetal DNA molecules appeared to be shorter than the maternal DNA molecules [14]. Another approach for measuring the size of cfDNA molecules was based on electron microscopy [17]. However, neither qPCR nor electron microscopy is able to scale up for a genome-wide survey of the size characteristics of cfDNA.

With the advent of next-generation sequencing, it was demonstrated that fragment size could be deduced at single-base resolution for cfDNA molecules originating from virtually any genomic location [18,19,20,21]. In these studies, cfDNA molecules were sequenced from both ends (i.e., paired-end sequencing). The paired-end reads were aligned to a reference genome to determine the outermost coordinates of a fragment such that the whole size of that fragment was determined by the subtraction of outermost coordinates. For cfDNA molecules larger than the read length, a part of the sequence at the middle of such a cfDNA fragment is not present in the sequencing results. Hence, certain genomic and epigenetic information of cfDNA molecules would be missing when using next-generation sequencing. Another limitation is that next-generation sequencing is not able to effectively sequence those cfDNA molecules above 500 bp [22,23]. One possible reason would be that DNA amplification, involved in the sequencing library preparation and/or sequencing cluster generation in a flow cell, might favor shorter DNA templates (e.g., <200 bp) over longer DNA templates (e.g., >500 bp). During the DNA amplification, a longer extension time might be required to synthesize the daughter strands, and the longer DNA templates might have a higher probability of forming secondary structures [24].

Recently, Yu et al. demonstrated that the use of single-molecule sequencing allowed the direct determination of cfDNA molecule sizes. Single-molecule sequencing is a long-read sequencing technology that is able to interrogate each nucleotide present in an entire cfDNA molecule [25]. The single-molecule sequencing has some intrinsic advantages over short-read sequencing, for example, allowing the interrogation of fragmentomics (e.g., sizes and ends), genetic, and epigenetic information (e.g., variants and CpG methylation) of a cfDNA molecule.

### 2.2. cfDNA Size Profile

**Figure 2:**
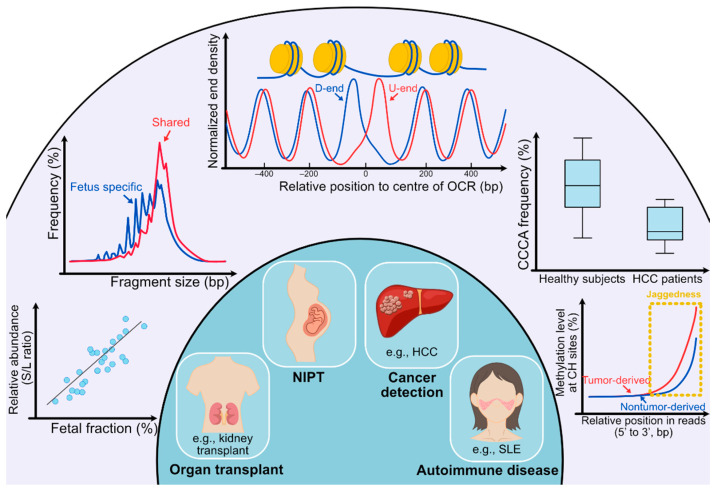
**Clinical utilities of cell-free DNA fragmentomics. (Outer circle)** In the outer circle, several analyses of cfDNA molecules are listed. From left bottom to right bottom, the first analysis shows the analysis concerning preferred ends. ‘S’ stands for preferred end sites for short fragments (60–155 bp) and ‘L’ stands for preferred end sites for long fragments (170–250 bp). A positive correlation can be observed between the relative abundance of fragments with set S versus set L preferred end sites (denoted as the S/L ratio) and the fetal DNA fraction. The second analysis shows that the size of fragments with fetal-specific single nucleotide polymorphisms (SNPs) is generally shorter than that of shared alleles. The third analysis is for deducing tissues of origin. Fragments released from different tissues show diverse nucleosomal positioning patterns, which can be represented by calculating the cumulative difference in normalized end density of U-ends and D-ends with 60 bp away from the center of the open chromatin regions (OCR) in different directions. In the fourth analysis, the frequency of CCCA, which is the end motif of the highest frequency in healthy human subjects, significantly decreases in HCC patients. For the fifth analysis, the methylation level at CH sites gradually increases as the CH sites are closer to the 3’ end of the fragments, and the increase in tumor-derived DNA molecules overtakes fragments carrying wild-type alleles. Such an increase in methylation level is because of the presence of jagged ends on plasma DNA molecules, which incorporates methylated cytosines during the DNA end-repair process. The tumor-derived DNA molecules would carry relatively more and longer jagged ends. **(Inner circle)** In the inner circle, four main applications (organ transplantation, NIPT, cancer detection, and autoimmune disease) are implicated by analyses of fragmentation patterns of cfDNA molecules.

cfDNA fragmentations nonrandomly occur in blood circulation, depending on the tissues of origin. Lo et al. demonstrated that maternal and fetal DNA molecules possessed characteristic size patterns in the maternal plasma of a pregnant woman [18] using massively parallel paired-end sequencing. Both maternal and fetal DNA molecules displayed a major peak at 166 bp in the respective size distributions. The size profile of fetal cfDNA molecules was relatively located on the left side of that of maternal cfDNA, with a secondary predominant peak at 143 bp, suggesting that fetal DNA molecules were relatively shorter than the maternal DNA molecules (Figure 2). The shortening of fetal cfDNA molecules was speculated to be related to the trimming of approximately 20-bp linkers that might be susceptible to DNA nuclease degradation because of a lack of the protection of histone cores [18]. Another noticeable feature was that both maternal and fetal cfDNA exhibited a series of 10-bp periodicities in those smaller cfDNA molecules, but the fetal cfDNA had enhanced amplitudes across the periodicities. The 10-bp periodicities are reminiscent of DNA helix interacting with nucleosome core in 10 bp per turn. Hence, the fragmentation of cfDNA would occur in association with nucleosome structures relating to tissues of origin. Many other studies also reproduced the characteristic size profiles of cfDNA in plasma of subjects with different pathological or physiological statuses. For example, in healthy human subjects, cfDNA molecules generally also showed a major peak at 166 bp with a series of 10-bp periodicities at a size shorter than 143 bp [2,5,18,19,21,26]. In the plasma of patients with organ transplantation, the non-hematopoietically-derived cfDNA molecules were shorter than background DNA molecules mainly of hematopoietic origin [19]. In the plasma of patients with cancer, the tumor-derived cfDNA molecules were demonstrated to be generally shorter [5,9,10,27].

Yu et al. took advantage of the fact that the fetal DNA was shorter than maternal DNA molecules for measuring fetal fraction and detecting fetal aneuploidies [7,8]. The higher the fetal DNA fraction, the more short cfDNA molecules would be present in the maternal plasma. The affected chromosome in a trisomic fetus would result in the increase in extra shortening or lengthening of cfDNA molecules originating from that chromosome in maternal plasma, depending on whether or not the affected chromosome was subjected to the copy number gain or loss. On the basis of this principle, the sensitivity in fetal trisomy 21 and trisomy 18 detection was 100%, with a specificity of 100% [7], which was comparable with the performance of the count-based approach for fetal aneuploidy detection [1]. Of note, combining the size- and count-based analysis could further determine the fetal or maternal origin of the copy number aberrations, including microduplications or microdeletions seen in maternal plasma, leading to a better interpretation of NIPT results [8]. The detection of fetal de novo mutations, which was rare and extremely challenging in the overwhelming maternal background DNA (i.e., a typically low positive predictive value (PPV) issue), had been facilitated using a size-based novel filtering algorithm by which the true de novo mutations from the fetus was required to be associated with shorter sizes [28], resulting in a two-orders-of-magnitude-higher positive predictive value.

Hence, the use of sizes of cfDNA molecules improved the overall performance of NIPT. In parallel, it was demonstrated that the use of size properties of tumor-derived cfDNA in plasma of patients with cancer could inform the tumor DNA fraction and facilitate cancer detection [5]. Many lines of evidence suggest that a better performance in cancer detection using liquid biopsy would possibly be achieved. Jiang et al. illustrated that taking advantage of size properties (i.e., shortening of tumor-derived DNA) would enable high performance of detecting tumor-derived somatic mutations in patients with hepatocellular carcinoma, with a PPV of 85%. Marass et al. suggested the feasibility of using fragment sizes to differentiate clonal hematopoiesis from tumor-derived mutations in cell-free DNA [29]. Mouliere et al. reported that the enhanced detection of circulating tumor DNA was achieved by making use of the amount of cfDNA molecules, together with fragmentation features including proportion (P) of fragments in defined size ranges of P(160–180), P(180–220), P(250–320), and the amplitudes of 10-bp periodicities. Receiver operating characteristic (ROC) curve analysis showed that the differentiation of cancer patients and healthy subjects was improved with an area under the ROC curve (AUC) of 0.91 [9].

On the basis of the realization of the size characteristics of cfDNA, Cristiano et al. further developed an approach to interrogate the size patterns of cfDNA across the genome. It was found that cfDNA size profiles of healthy individuals were suggestive of nucleosomal patterns of white blood cells, whereas patients with cancer had aberrations present in size profiles. Analyzing fragmentation profiles of 236 patients with breast, colorectal, lung, ovarian, pancreatic, gastric, or bile duct cancer, and 245 healthy individuals, a machine learning model utilizing genome-wide size features had sensitivities ranging from 57% to >99% at a specificity of 98% [30]. Size profiles facilitated the determination of the tissue of origin of the cancers to a limited number of sites in 75% of cases, further validating that the fragmentation of cfDNA bears the information relating to the tissues of origin.

In addition, researchers reported that patients with systemic lupus erythematosus (SLE) were found to exhibit a skewed size profile with a significant increase in short fragments that could potentially serve as a hallmark for the SLE disease activity [26]. Thus, the use of fragmentation of cfDNA molecules would signify a wide range of pathological statuses, such as cancer and autoimmune diseases.

Most recently, with the use of single-molecule real-time sequencing (PacBio SMRT-seq), Yu et al. unveiled a large population of long cfDNA molecules (>500 bp) present in the plasma of pregnant women, with a median of 15.5%, 19.8%, and 32.3% for the first, second, and third trimesters, respectively. The longest fetal-derived cfDNA reached up to 23,635 bp [25]. Both fetal and maternal DNA molecules presented more long DNA as the maternal gestational age advanced. The proportion of long cfDNA would significantly decrease in pregnancies with preeclampsia, potentially providing the biomarker for preeclampsia. The differentiation of pregnancies with and without preeclampsia on the size-based metric by single-molecule sequencing appeared to be superior to the short-read sequencing (Illumina, San Diego, CA, USA) (AUC: 1 versus 0.7) [25]. These results suggested that the accurate profiling of the full-size spectrum of cfDNA molecules would improve the diagnostic performance. The use of long cfDNA molecules that were not appreciated before would open up new possibilities for NIPT. Yu et al. further demonstrated that as a long molecule would contain a series of CpG sites, taking advantage of its methylation pattern enabled the determination of the tissue of origin for individual plasma DNA molecules. In other words, the methylation pattern in a long plasma DNA molecule served as an intrinsic ‘molecular barcode’, indicating cell identity.

The generation of cfDNA molecules was reported to be associated with enzymatic processes [18,31,32,33]. The deficiency of DNASE1L3 (Deoxyribonuclease 1 Like 3) would lead to an increase in both short (<120 bp) and long cfDNA (>250 bp) involving multiple nucleosome units (di-, tri-, and tetra-nucleosomal sizes) in mice and humans, whereas the frequency of the major peak of 166 bp decreased [31]. Hence, the DNA nuclease activity would be one of the factors governing the size characteristics of cfDNA molecules.

## 3. End Signature of cfDNA

### 3.1. Preferred Ends

Characteristic size profiles of cfDNA molecules from different tissues convincingly indicate that the fragmentation of cfDNA molecules is nonrandom depending on the cell identities. Using ultra-deep sequencing of plasma DNA (a mean of 230× haploid genome coverage), Chan et al. showed that a subset of the genomic coordinates was preferentially cleaved in the generation of plasma DNA of pregnant women, forming so-called ‘preferred ends’ [28]. The result showed that 25% of cfDNA fragments had at least one counterpart sharing the same end sites. Such preferred ends exhibited selectivity for fetal- and maternal-derived DNA in maternal plasma. Thousands of genomic positions related to fetal-specific cfDNA as well as maternal-specific cfDNA were identified. Plasma DNA molecules originating from fetal-specific preferred genomic sites carried the properties of fetal cfDNA. For instance, those cfDNA molecules tended to be shorter in size and correlated with fetal DNA fraction (Figure 2) [34].

The clusters of preferred ends were shown to be in line with nucleosomal patterns along the genome (35), also depending on tissue specificity. Moreover, liver-specific preferred ends were identified in the plasma of patients with liver transplantation [35]. Of note, the AUC for distinguishing hepatocellular carcinoma (HCC) patients from healthy subjects reached 0.88 using the ratio of tumor-associated and nontumor-associated preferred ends [35], suggesting that the preferred ends could serve as a biomarker for cancer. The large number of preferred end coordinates available across the genome potentially makes it possible to detect cancer at its early stage. Moreover, the detection of tumor-associated plasma DNA ends may offer a cost-effective means of capturing evidence of the presence of cancer through a liquid biopsy assessment.

### 3.2. End Motifs of cfDNA

In addition to the ending sites, researchers are actively exploring the compositions for a number of nucleotides proximal to the 5’ end of a cfDNA molecule, referred to as end motifs. Serpas et al. studied 4 nucleotides proximal to 5′ end (i.e., 4-mer end motifs, a total of 256 motifs) in mice with different genotypes of DNA nuclease knockouts [31]. The frequency of the most common motif CCCA in plasma of wild-type mice was dramatically decreased in mice with *Dnase1l3* deletion. The frequency of the top 6 motifs in plasma of wild-type mice showed a significant reduction in *Dnase1l3* deletion mice, including CCCA, CCTG, CCAG, CCAA, CCAT, and CCTC, for which all started with CC, accounting for 7.43% and 4.22% of cfDNA molecules in wild-type and *Dnase1l3* knockout groups, respectively. In contrast, the knockout of *Dnase1* appeared to have no clearly observable effect [31]. Hence, these data suggested that DNASE1L3 would be one of the major players in generating ‘CC’ ends of plasma DNA molecules. Interestingly, *Dnase1l3^−/−^* mice (both copies of the *Dnase1l3* gene are deactivated) carrying *Dnase1l3^+/^*^−^ fetuses (one copy of the *Dnase1l3* gene is active) could lead to a partial restoration of normal frequencies of end motifs for both maternal and fetal DNA, but with an enhanced restoration on fetal DNA molecules. Thus, the DNASE1L3 acts on maternal DNA fragmentation in either a systemic or local manner. Plasma end-motif analysis provided a window for observing the link between cfDNA fragmentation and nuclease activities. Interestingly, the DNASE1L3-cutting signature (i.e., ‘CC’ end motif) was reported to be well generalized in the plasma of human subjects with DNASE1L3 deficiency which causes familial monogenic SLE with childhood onset. In addition, adeno-associated virus-based transduction of *Dnase1l3* into *Dnase1l3*-deficient mice could restore the end-motif profiles to those normally present in the plasma DNA of wild-type mice [36]. These studies indicate that the analysis of nuclease-associated cutting signatures could be a powerful diagnostic tool for disease detection and therapeutic response monitoring.

Han et al. further elucidated the roles of DNASE1 (Deoxyribonuclease 1), DNASE1L3, and DFFB (DNA Fragmentation Factor Subunit Beta) across different knockout mouse models using in vitro incubations of blood with EDTA or heparin. The anticoagulant EDTA inhibited both DNASE1 and DNASE1L3 activities by chelating divalent cations [37,38]. Heparin, which has a stronger binding affinity to histones than DNA molecules and repels DNA off from histones, was known to enhance DNASE1 activity while inhibiting DNASE1L3 [37]. The study demonstrated that a stepwise process of DNA fragmentation would occur during the generation of circulating cfDNA. As a simplistic view, cfDNA was initially generated intracellularly with DFFB that favored generating A-end fragments, followed by circulating DNASE1L3 and DNASE1 which favored generating C-end fragments and T-end fragments, respectively [32].

Interestingly, the end motif CCCA, the most frequent end motif in plasma DNA of healthy human subjects, was found to be decreased in patients with HCC (Figure 2) [39]. The mRNA expression of DNAES1L3 in HCC tumors was shown to be downregulated, compared with normal adjacent non-tumoral tissues [39], which might be reflective of a reduction in DNASE1L3. The motif alterations in patients with HCC were, to some extent, reminiscent of the reduced DNASE1L3 activities acting on cfDNA fragmentations learned from the mouse model. Interestingly, the downregulation of DNASE1L3 expression was commonly seen across various cancer types such as colorectal cancer, lung cancer, nasopharyngeal carcinoma, and head and neck squamous cell carcinoma [39], supporting the potential decline of DNASE1L3 in patients with cancers. The holistic use of 256 motifs could provide a tool for detecting multi-cancers. For example, the use of the motif diversity score could achieve an AUC of 0.86. Many other groups further demonstrated the potential clinical applications in oncology by making use of plasma DNA end motifs [40]. The tissue specificity of plasma DNA end motifs was revealed in liver-derived molecules and fetal DNA molecules based on data generated from patients with liver transplantation and pregnant subjects [39].

### 3.3. End Orientation Analysis and Nucleosome Footprint Analysis

cfDNA size profiles and preferred ends are suggestive of the cfDNA fragmentation related to nucleosome structures [18,28]. Nucleosomal positions within the human genome exhibit positional variation among cell types [34,41,42]. Lo et al. postulated that the 166-bp cfDNA molecules represented fragments containing the nucleosome core with the linker [18]. In contrast, the 143-bp cfDNA molecules were postulated to represent fragments containing the nucleosomal core without the linker. On the basis of this idea, Straver et al. computationally compiled a “nucleosome track” by pooling maternal plasma DNA sequencing data from multiple cases [43]. The frequency of reads with starting sequences within regions 73 bp upstream and downstream of the deduced nucleosome center showed a positive correlation with the fetal DNA fraction [43]. Outside of the context of pregnancy, Snyder et al. also explored the nucleosomal footprints of plasma DNA across patients with different cancers. They used a metric called the window protection score, which was defined as the number of molecules spanning a particular genomic window (120 bp) minus those molecules with an endpoint residing within the window [44]. By employing machine learning, Ulz et al. proved the feasibility of using read-depth patterns of plasma DNA surrounding transcriptional start sites to reflect gene expression levels [45].

To simultaneously deduce the relative contributions from different tissues, Sun et al. took advantage of the differential frequencies corresponding to the orientation of the upstream and downstream ends of cfDNA molecules in relation to open chromatin regions, referred to as orientation-aware plasma cell-free DNA fragmentation (OCF) analysis. Such orientation-aware fragmentation patterns depended on tissue-specific open chromatin regions, facilitating the quantitative measurement of the relative contributions of various tissues toward the plasma DNA pool (Figure 2). OCF analysis opened up many new possibilities for developing applications in noninvasive prenatal testing, organ transplantation monitoring, and cancer liquid biopsies [46]. Taken together, these studies concluded that plasma DNA cleavages or breakages that cluster around genomic coordinates are in relation to nucleosome positionings and open chromatin domains.

### 3.4. Jagged Ends of cfDNA

The fragmentomic features discussed above, including fragment sizes, preferred ends, and end motifs, were based on sequencing results generated from the double-stranded cfDNA molecules after the completion of DNA end-repair. The blunt ends of cfDNA molecules are formed as a result of the end repairing step by which the 5’ single-stranded protruding ends are filled up, and the 3’ single-stranded protruding ends are trimmed off when these protruding ends are present in the plasma DNA molecules. Hence, the existence of single-stranded DNA at the ends of a double-stranded cfDNA molecule (herein referred to as jagged ends) has been missed for many years. To explore such a gap in our knowledge, Jiang et al. developed approaches to detect the jagged ends of plasma DNA molecules by introducing the differential methylation signals into the complementary strand of the single-stranded DNA present at 5’ ends [47]. For example, during the DNA end-repair process with the presence of methylation cytosines, the methylated cytosines could be introduced into the extending strand opposite a single-stranded jagged end. As non-CpG sites in the human genome are nearly unmethylated, the increase in methylation levels across non-CpG sites proximal to the 3’ end of the newly generated strand suggested the presence of jagged ends. Compared with sonicated DNA molecules, plasma DNA molecules bore a significantly higher level of jaggedness. Furthermore, this approach allowed the deduction of the exact length of jagged ends on the basis of the principle that the starting point of a jagged end could be demarcated by the pattern of one unmethylated status immediately followed by the other methylated status at two consecutive cytosines. It turned out that 88% of plasma DNA molecules would carry jagged ends. Interestingly, the fetal DNA and tumoral DNA molecules were characterized with higher jaggedness, compared with background DNA molecules predominantly of hematopoietic DNA in pregnancies and cancer patients, respectively (Figure 2). Moreover, the aberration in plasma DNA jaggedness could serve as a biomarker for human subjects with either familial or sporadic SLE, for which an increase in jaggedness was often observed [48].

In a recent study [48], jagged-end lengths of plasma DNA were reported to be associated with DNASE1, DFFB, or DNASE1L3 activities, depending on nucleosomal structures. DNASE1 would generate jagged ends across a wide size spectrum, regardless of nucleosomal core or linker DNA. DFFB would tend to generate jagged ends in linker DNA between two nucleosomes, with a blunt end or relatively shorter jagged ends. In addition, DNASE1L3 played a more pronounced role in producing jagged ends of plasma DNA, preferentially introducing jagged ends to DNA molecules involving multi-nucleosomes. It will be important to synergistically explore different technologies to study the emerging property of cfDNA molecules across different disease models, such as a ligation-based assay [49].

## 4. Characteristic Size Patterns for Extrachromosomal Circular DNA (eccDNA)

In sharp contrast to the linear cfDNA molecules presenting one major peak at 166 bp with a series of smaller peaks at intervals of approximately 10 bp, the size profile of eccDNA molecules is distinct, displaying two major peaks at 202 bp and 338 bp with sharp 10-bp periodicities [50]. It implied that the biological processes of generating eccDNA molecules might be different from linear cfDNA. The generation of eccDNA might also involve nucleosomal structures. For example, Sin et al. postulated that the size peak of 202 bp might be related to the size of one nucleosome core and two linkers, while a size peak of 338 bp might consist of two nucleosome cores and two linkers [50,51]. Surprisingly, the clearance rate between eccDNA and linear cfDNA molecules did not appear to be clearly different in plasma of pregnant women [51], which seems paradoxically counterintuitive to some previous studies speculating that a more stable structure of eccDNA would survive longer than linear DNA [52]. More studies would be warranted to gain more biological insights regarding the functionality and robustness of eccDNA in blood circulation.

The fetal-derived eccDNA was tended to be shorter than the maternal-derived ones [50,51]. A lower methylation level of fetal-derived eccDNA molecules was observed [51]. Such two types of characteristics of fetal eccDNA were reminiscent of that in linear cfDNA molecules. Moreover, eccDNA fragments at the second peak cluster (~338 bp) tended to be more hypermethylated when compared with the first peak cluster (~202 bp). One potential explanation could be that the methylation would tighten the nucleosome package [53], increasing the protection from nuclease degradation.

In addition to eccDNA molecules, a substantial portion of mitochondrial DNA (mtDNA) in plasma could potentially present as its original circular form (i.e., ~16.5 kb), depending on the tissue specificity [54,55]. Ma et al. elucidated the coexistence of linear and circular forms of mtDNA in plasma based on the digestion of plasma DNA with a restriction enzyme (RE) [54]. A digested fragment of mtDNA carrying RE-cleaved end signatures at both ends would be prone to derive from a circular form, whereas a fragment of mtDNA carrying none of or one RE-cleaved end signature could be likely attributed to a linear form. Interestingly, when studying the linear and circular forms of mtDNA in patients with liver transplantation, it was found that the majority of mtDNA derived from the liver were of the linear form (91%), whereas the majority of mtDNA derived from the hematopoietic system were of the circular form (88%). Hence, the nonhematopoietic and hematopoietic systems would shed different forms of mtDNA into the blood circulation, which had been further confirmed in the surrogate pregnancy and the bone marrow transplantation models [55].

## 5. Concluding Remarks

Fragmentation patterns of cfDNA molecules have attracted a lot of recent research interest, including fragment sizes, nucleosome relationships, endpoints, end motifs, and topological forms, forming a field of cfDNA fragmentomics. Fragmentomic features of cfDNA molecules bear a wealth of molecular information pertaining to the tissues of origin, paving the crucial foundation for exploring and developing potential diagnostic tools based on cfDNA fragmentomics. Such development could accelerate the development of high-performance diagnostic tools for NIPT, cancer detection, monitoring of organ transplantation, as well as detection of other diseases (e.g., autoimmune diseases) (Figure 2).
